# Draft Genome Sequence of a Highly Resistant Streptococcus pneumoniae Serotype 15A Strain Isolated from Blood

**DOI:** 10.1128/genomeA.00167-18

**Published:** 2018-04-19

**Authors:** Revathy Arushothy, Norazah Ahmad, Fairuz Amran, Rohaidah Hashim, Nazirah Samsuddin, Che Roslina Che Azih

**Affiliations:** a Bacteriology Unit, Infectious Diseases Research Centre, Institute for Medical Research, Jalan Pahang, Kuala Lumpur, Malaysia

## Abstract

After the introduction of the pneumococcal conjugate vaccine in Malaysia in recent years, the emergence of nonvaccine serotypes is of concern, particularly the antibiotic-resistant strains, with an increase specifically in serotype 15A. Here, we report the draft genome sequence of Streptococcus pneumoniae strain SS40_16, isolated from the blood sample of a 19-month-old female in 2016. SS40_16 is a multidrug-resistant strain with resistance to penicillin (MIC, ≥2 µg/ml), tetracycline, and trimethoprim-sulfamethoxazole. The strain belongs to serotype 15A and sequence type 1591 (ST1591).

## GENOME ANNOUNCEMENT

With the introduction of the pneumococcal conjugate vaccine in recent years, the emergence of nonvaccine serotypes is of concern, particularly the antibiotic-resistant strains. There has been an increase in the prevalence of serotype 15A, with many isolates showing triple resistance to macrolides, tetracyclines, and penicillin, implying that the surface polysaccharides of serotype 15A support virulence (1).

Here, we report the draft genome sequence of Streptococcus pneumoniae strain SS40_16 isolated in 2016 from a blood sample from a 19-month-old female from a government hospital in Malaysia. The strain belongs to serotype 15A and sequence type 1591 (ST1591) (as determined by PubMLST [https://pubmlst.org/]). SS40_16 is a multidrug-resistant strain with resistance to penicillin (MIC, ≥2 µg/ml), tetracycline, and trimethoprim-sulfamethoxazole.

The genome was sequenced using an Illumina MiSeq system (Illumina, San Diego, CA, USA), with 2 × 301-bp paired-end chemistry. *De novo* genome assembly was performed with SPAdes version 3.9.0. The genome of SS40_16 was assembled into 128 contigs, with a total length of 2,121,989 bp. It has 277,158 paired reads and 39.42% G+C content (2).

The protein-coding genes were predicted using Prodigal version 2.60, in which 2,049 protein-coding genes were predicted. ARAGORN version 1.2.34 was used for tRNA gene prediction, resulting in 37 predicted tRNAs, and RNAmmer version 1.2 was used for rRNA gene prediction, resulting in 3 predicted rRNAs (3). Genome annotation on predicted genes was carried out using BLASTN version 2.2.25+, with similarity searches against the Swiss-Prot database, with a total of 55% genes successfully annotated (4). BLASTN was used for sequence similarity search/gene function prediction, and 100% similarity to Streptococcus pneumoniae ATCC 700669 was found (5).

The SS40_16 genome contains tetracycline, fluoroquinolone, and multidrug resistance efflux pump genes. It also contains a vancomycin tolerance locus; however, the strain is sensitive to vancomycin.

### Accession number(s).

The nucleotide sequence of the Streptococcus pneumoniae SS40_16 genome has been deposited in DDBJ/EMBL/GenBank under accession no. PEKA00000000.
